# Early-Onset Neonatal Sepsis: Role of C-Reactive Protein, Micro-ESR, and Gastric Aspirate for Polymorphs as Screening Markers

**DOI:** 10.1155/2021/1544553

**Published:** 2021-12-02

**Authors:** Sukhdeep Kaur, Kunwarpal Singh

**Affiliations:** ^1^Department of Pediatrics, Sri Guru Ram Das Institute of Medical Sciences and Research, Vallah, Sri Amritsar, Punjab, India; ^2^Department of Radiodiagnosis and Imaging, Sri Guru Ram Das Institute of Medical Sciences and Research, Vallah, Sri Amritsar, Punjab, India

## Abstract

**Introduction:**

Early-onset neonatal sepsis is a major cause of morbidness and death in newborn children. Its timely diagnosis is usually a challenge in developing countries like India.

**Aim:**

To study the efficacy of C-reactive protein (CRP), micro-ESR, and gastric aspirate for polymorphs in the diagnosis of early-onset neonatal sepsis.

**Materials and Methods:**

This study included sixty term and preterm children, inborn and referred cases. The children who presented before day seven of life with clinical suspicion of sepsis or who were at high risk of developing sepsis were included. These were further investigated. Significant values for screening tests were taken as C − reactive protein > 0.6 mg/dl, micro-ESR—after 1 hour, fall in the column of blood in capillary tube was measured, and result was taken as mm fall in 1 hr, and gastric aspirate for polymorphs > 5 polymorphs/HPF. Sepsis screen positive result was 2 or more positive tests. The statistical evaluation was done using Fisher, and ANOVA tests using SPSS 20.0 version.

**Results:**

Sixty children were included in the study with forty as the referred ones. Most of them had tachypnea (45%). CRP showed high sensitivity, whereas micro-ESR and gastric aspirate for polymorphs showed high specificity.

**Conclusions:**

Neonatal sepsis screening is required for the detection of infection as the blood culture report may not be positive in all the cases, and even if positive, the result takes few hours. CRP showed high sensitivity, whereas micro-ESR and gastric aspirate for polymorphs showed high specificity independently as well as when combined.

## 1. Introduction

Neonatal septicemia remains a significant cause of morbidity and mortality in newborn infants. The incidence of neonatal sepsis varies between 11.0 and 24.5/1000 live births in India [1]. Bacterial infections often present a diagnostic challenge in the resource-poor setting of most developing countries.

Successful treatment depends on the early initiation of antibiotics, but early diagnosis of neonatal infections is difficult because clinical signs are nonspecific and may initially be subtle. Respiratory distress, apneic spells, episodes of bradycardia, feeding intolerance, lethargy, and temperature instability, as well as minor changes on physical examination or in clinical status, are some of the conditions that suggest a possible neonatal infection and need sepsis evaluation.

The clinical signs of early-onset sepsis are usually apparent in the first hours of life; 90% of infants are symptomatic by 24 hrs of age. Respiratory distress is the most common presenting symptom. Respiratory symptoms can range in severity from mild tachypnea and grunting with or without supplemental oxygen requirement to respiratory failure. Other less-specific signs of sepsis include irritability, lethargy, temperature instability, poor perfusion, and hypotension. Gastrointestinal symptoms can include poor feeding, vomiting, and ileus. Meningitis may present with seizure activity, apnea, and/or depressed sensorium. So, the early-onset disease can manifest as asymptomatic bacteremia, generalized sepsis, pneumonia, and/or meningitis [2].

Although the onset of illness is often inconspicuous, the clinical course may be alarmingly fulminant, leading to septicaemic shock, disseminated intravascular coagulation, and death within hours of the onset of clinical manifestations. Sepsis evaluations are performed, and empiric antibiotic therapy is begun even when there is a remote possibility of infection. A wide variety of hematological and biochemical markers have been investigated for the evaluation of clinical sepsis. Hematological tests like TLC, TNC, INC, I/T ratio, and morphological or degenerative changes in neutrophil and platelet count have been studied either singly or in combination. Results of white cell counts and ratio varied widely across studies with sensitivity and specificity ranging from 17.0% to 90.0% and 31.0% to 100% [3].

Acute-phase proteins are produced principally by the liver as part of an immediate inflammatory response to infection or tissue injury. The most extensively used and investigated acute phase reactant is C-reactive protein (CRP). CRP as a diagnostic marker in neonates has higher sensitivity and specificity than total neutrophil count and I/T ratio. Serial measurements at 24 and 48 hrs after the onset of illness considerably improve the sensitivity (82.0% and 84.0%, respectively). Serial CRP showed very high predictive values for the diagnosis of neonatal sepsis and was better than those of leucocyte indices of CBC [4]. During the first three days of life, CRP, leucopenia, and neutropenia were comparably good tests while after three days of life; CRP was the single best test in early detection of neonatal septicemia [5].

Jaswal et al. studied the C-reactive protein levels to evaluate the duration of antibiotics in 50 consecutive neonates with suspected septicemia. The negative predictive value of serial CRP was 100% in deciding the duration of antibiotics therapy in suspected neonatal septicemia up to 7 days [6].

Bacterial infections often present a diagnostic challenge in the resource-poor setting of most developing countries partly because symptoms are nonspecific and also because most healthcare facilities lack adequate laboratory facilities. With a neonatal mortality rate of 44 per thousand live births, India contributes to 30.0% of neonatal deaths worldwide. Neonatal septicemia is the most common indication for the admission of neonates brought from the community to subdistrict, district, and tertiary care hospitals [7].

Because bacterial sepsis can be rapidly progressive, the physician must be alert to signs and symptoms of possible infection and initiate diagnostic evaluation and empirical therapy at an early stage. Gupta et al. did a clinical study in 51 cases of neonatal klebsiella septicemia. They documented that lethargy, feeding problems, abdominal distension, respiratory distress, hypothermia, apnea, and irritability were the most common presenting features in these patients [8]. The mean age of onset of clinical symptoms was 5.7 ± 2.2 days. The presence of respiratory symptoms, haemorrhage, septicemic shock, and leucopenia were the bad prognostic factors. Early-onset sepsis manifests from birth to 7 days, usually less than 72 hrs, and the responsible organism is frequently from maternal genital flora.

Philip and Hewitt evaluated the ability of a sepsis screen involving 5 tests to discriminate infants with early-onset sepsis from noninfected neonates. They used TLC < 5000/cu mm, I/T ratio (>0.2), latex CRP positive (>0.8 mg/dl), micro − ESR > 15 mm in first hr, and latex haptoglobin positive (>25 mg/dl). They found 28 of 30 cases (93.0%) of proven infections had positive screening defined as two or more abnormal tests [9].

It is now reasonable to consider CRP as a potential tool for antibiotic therapy and prophylaxis. This suggestion has moved a step closer to reality with the isolation of CRP-specific human complementary DNA probe and the discovery that the CRP gene is located on chromosome I as described by Whitehead et al. [10].

ESR is a nonspecific test of tissue damage. It is known to be elevated in collagenosis, cancer, infarction, and infection. Out of these, infection is the only one that is a common cause of illness or death in the neonatal period. The micro- or mini-ESR method is used in neonates because it requires only a few drops of capillary blood. Micro-ESR determination was first described by Landau and later by Smith Evans et al. evaluated micro-ESR in newborn infants for the first time. They found that normal values appeared slightly higher in the female infants although the range was approximately the same for both groups. The ninety-fifth percentile for both sexes was 6.0 mm per hour. The range of normal values for full-term infants was similar to that of the low-birth-weight infants. Among the 9 infants with serious infection included in their study, 8 showed elevated values of micro-ESR. In addition, a normal value of ESR observed in idiopathic respiratory distress syndrome helped differentiate this entity from the infectious process [11, 12].

The bedside micro-ESR level test showed significance in the diagnosis of neonatal sepsis for better management in the NICU in the study done by Manandhar and Basnet. Microerythrocyte sedimentation level was elevated in 33.3% of babies. The elevated microerythrocyte sedimentation level was seen with sepsis types and C-reactive protein [13].

Ahmed et al. analyzed various parameters of sepsis screen singly and in combination to formulate a guideline for diagnosis of neonatal sepsis. CRP was positive in 24/28 (85.7%) cases of group A (proven sepsis), and 58/72 (80.5%) cases of group B (probable sepsis) and had a specificity of 95.0%. Absolute neutrophil count was the second most sensitive test having a sensitivity of 71.4% for group A and 63.9% for group B and 88.0% specificity for group A. Sensitivity of gastric aspirate for polymorphs and platelet count was 71.4% and 64.3%, respectively. A set of investigations including CRP, TLC, ANC, thrombocytopenia, cytoplasmic vacuolization in the neutrophils, and gastric aspirate for polymorphs are highly sensitive in the detection of culture-negative cases of neonatal sepsis. Moreover, a combination of these tests enhances their sensitivity [14].

Though the commencement of illness is not noticeable, the clinical course may be bad and may lead to shock, DIC, or demise with few hours. Detailed evaluation of sepsis is done, and antibiotics were started even when there are even remote chances of infection. A large number of screening markers have been investigated for sepsis evaluation including TLC, I/T ratio, and morphological or degenerative changes in neutrophil and platelet count either singly or combined together.

## 2. Materials and Methods

The study was conducted on sixty neonates at the neonatal intensive care unit attached to the Department of Pediatrics and Neonatology of a 242-bedded hospital located in North India. The neonatal intensive care unit is well equipped with modern facilities such as advanced modes of ventilation and parenteral nutrition along with different categories of beds and wards for different social strata. The admission to NICU comprises of outside as well as inborn neonates. The study was a prospective study conducted for a year. The babies, who had the clinical symptoms and signs of suspected neonatal sepsis/high-risk factors for developing the sepsis, were included. Informed written consent was taken from the parents/attendants of the admitted neonates. The initial evaluation by history, examination, and investigation was carried out on each. Neonates who had received antibiotics were excluded from the study. Sepsis screening was done at admission for all neonates enrolled in the study. The criteria for selection were as follows.

Each patient was subjected to detailed history and physical examination. Blood samples were taken at admission and subjected to the following tests: total leucocyte count (TLC), C-reactive protein (CRP), micro-ESR (*μ* ESR), and gastric aspirate cytology for polymorphs (GAC). The blood sample for blood culture and sensitivity was collected at the same time. Following this, the decision to start antibiotic therapy was based on the combination of clinical signs, obstetric risk factors, and sepsis screen. Furthermore, the sepsis screen was repeated whenever new clinical signs of infection developed. The samples were collected in an EDTA vial for TLC and a plain vial for CRP.

Under strict aseptic measures, samples for blood culture and sensitivity were collected. Gastric aspiration was sent for cytology in plain sterilized tubes. Total leucocyte count was measured by the manual method using the Neubauer chamber and using an electronic cell counter.

### 2.1. Gastric Aspirate for Polymorphs

The gastric aspirate was obtained by infant feeding tube within 12 hrs of life in a neonate and put in a plain vial. One drop of gastric aspirate was mixed with one drop of methylene blue on a slide and covered with a coverslip. The slide was seen under the microscope for polymorphs/HPF.

### 2.2. Micro-ESR

Micro-ESR was determined in NICU by filling a microhematocrit tube with capillary blood and placing vertically for 1 hour, before measuring the distance from the top of the tube to the meniscus of the red blood cell column. The sample was taken in 75 mm long with an internal diameter of 1.1 mm and an external diameter of 1.5 mm heparinized capillary tube and was allowed to stand vertically sealing the lower end of the capillary tube. After 1 hour, a fall in the column of blood in the capillary tube was measured, and the result was taken as mm fall in 1 hr.

### 2.3. Blood Culture

Blood culture sample was collected from venipuncture under aseptic measures, cleaning the skin with spirit-betadine-spirit and collected in a 2 cc syringe and then transferred to BacT/ALERT PF bottle (20 ml) using another sterile needle.

### 2.4. Statistical Analysis

The statistical analysis was done to calculate sensitivity, specificity, negative predictive value, positive predictive value, and accuracy of various sepsis screening markers mentioned in the study. *p* values were also calculated. Analysis of variance (ANOVA) test was applied which is used to study whether the differences between groups of data are statistically significant. Fisher's exact test was also applied as it can be employed when sample sizes are small, although it is valid for larger sample sizes too. These statistical tests were applied using Statistical Package for the Social Sciences (SPSS) version 20.0.

Significant values for screening tests were taken as total leucocyte count of >25,000/<5000, C-reactive protein-positive (0.6 mg/dl), micro-ESR –2 or 3 + age in day's mm fall in 1 hr, and gastric aspirate for polymorphs > 5 polymorphs/HPF. Sepsis screen positive was two or more positive tests. The babies were started on IV antibiotics, while blood culture reports were awaited. The decision to continue antibiotics was taken depending upon the blood culture report. Babies were reassessed clinically, and a repeat sepsis screen was done if required.

## 3. Results

60 neonates admitted to the neonatal intensive care unit (NICU) attached to the Department of Pediatrics and Neonatology were enrolled in the study with risk factors and who fulfilled inclusion and exclusion criteria. Most of the neonates had presented with tachypnea. The neonates were admitted to the NICU with clinical symptoms and signs of tachypnea, refusal to feeds, lethargy, jaundice, abdominal distention, convulsions, cyanosis, fever, and oliguria.

CRP was positive in 39 (65.0%) of the neonates (Table 1). CRP was found to be the most sensitive parameter with a sensitivity of 87.5%. Micro-ESR was found to be highly specific (95.5%) but low sensitivity of 18.7%, PPV of 60.0%, and NPV of 76.3% (Table 2). Combination of sepsis screening markers showed high specificity (Table 3). Blood culture positivity was observed in 16 neonates. In the study, the most common organism grown in blood culture was Klebsiella in 10 (63.4%), followed by Staphylococcus aureus in 4 (25.0%), and Pseudomonas in 2 (11.6%) neonates.

## 4. Discussion

The major challenge is the prompt and accurate identification of infected infants. The task is frequently difficult because of the nonspecificity of clinical signs. A screening test should ideally detect all infected cases (high sensitivity) and high negative predictive value so that disease can be easily excluded, although essential for diagnosis and appropriate management blood culture results are not immediately available and their yield is low. In a study by Kumhar et al., the blood culture positivity was 42.0% [15].

Although not specific for neonatal sepsis, CRP has the highest sensitivity, specificity, high negative predictive value, and positive predictive value. In a cross-sectional study conducted on 50 neonates by Morad et al., CRP showed a sensitivity (89.5%), a specificity (66.7%), a PPV (92.5%), a NPV (60.0%), and a significant accuracy 86.0% (*p* value = 0.001) [16].

CRP estimation has now an established value as a marker of neonatal septicemia. In the present study, sensitivity, specificity, PPV, and NPV of CRP were 87.5%, 43.2%, 35.9%, and 90.5%, respectively.

Garland and Bowman assessed the usefulness of C-reactive protein (CRP) either alone or in combination with a full blood examination (FBE) and microbiology of gastric aspirate in predicting the diagnosis of neonatal sepsis compared with routinely available markers of infection. The sensitivity for CRP was 67.0%, and the negative predictive value (NPV) was 86.0%; FBE had a sensitivity of 63.0% and NPV of 80.0%; gastric aspirate had a sensitivity of 57.0% and NPV of 83.0% [17].

ESR had been a poor predictor of sepsis. This observation is in agreement with the findings of Shabbir et al. who observed that ESR had a sensitivity of 19.0% and a negative predictive value of 56.0% [18]. In the present study, micro-ESR had a specificity of 95.4%, a sensitivity of 18.7%, PPV of 60.0%, and NPV of 76.3%. Jaswal et al. found that the correlation between positive CRP, raised micro-ESR, and positive blood culture was significant (*p* < 0.005) [6]. Positive CRP and raised micro-ESR were similar to that reported by Bhartia et al. [19]. Similar results were observed in the present study. In a study conducted by Manandhar and Basnet in 75 babies, confirmed sepsis was observed in 17.3%, probable sepsis in 53.4%, and suspected sepsis in 29.3%. The micro-ESR level was normal in 66.7% of babies and elevated in 33.3% of babies. The mean micro-ESR level observed in this study was 9.32 ± 5.4 (2-18) mm in 1st hr [13].

Chekkali et al. studied the correlation of gastric aspirate polymorphs and acute phase reactants including micro-ESR, CRP, and band cell count with blood culture in early-onset neonatal sepsis. They concluded that CRP can be used for screening of early neonatal sepsis as its sensitivity, specificity, and positive predictive value was high, i.e., 72.2%, 82.1%, and 77.2%, respectively. Sensitivity, specificity, and positive predictive value of gastric acid polymorphs were 72.7%, 17.8%, 41.0%, and micro-ESR 54.5%, 67.8%, and 57.1%, respectively. CRP and band forms were more helpful than C-reactive protein and immature to total neutrophil ratio and micro-ESR in the screening of early neonatal sepsis [20]. In the present study, the combinations of tests had much higher specificity and negative predictive value. The combination of micro-ESR and gastric aspirate for polymorphs revealed 100% specificity and positive predictive value. The negative predictive value and accuracy were also high (75.8% and 76.6%, respectively) with significant *p* value (0.068). The combination of CRP + micro-ESR also revealed very high specificity (95.4%). However, both the above-mentioned combinations showed very low sensitivity of 12.5% and 18.7%, respectively.

Sharma et al. studied fifty clinically suspected cases of neonatal septicemia for evaluating the role of sepsis screen. Sensitivity and specificity of C-reactive protein test, micro-ESR, gastric aspirate cytology for polymorphs, and toxic granules in neutrophils were studied singly and also in combinations of two and three tests. Positive blood culture was obtained in only 20% of the cases, thereby, underlying the need for a sepsis screen in the diagnosis of neonatal septicemia, especially in the area where adequate microbiological facilities are lacking [21]. Similar results were observed in the present study.

Kaur and Singh studied the role of C-reactive protein and immature to total neutrophil ratio in early-onset neonatal sepsis. The authors concluded that C-reactive protein showed a high sensitivity while immature to total neutrophil ratio was found to be highly specific. The combination of C-reactive protein and immature to total neutrophil ratio showed a significant association with blood culture (*p* value 0.016) [22].

Klebsiella pneumoniae was found to be the most common organism responsible for neonatal septicemia in the present study. In a similar study by Chugh and Agarwal, it was observed that Klebsiella (68.8%) was the most common microorganism followed by Staphylococcus aureus in 11.1%, followed by Pseudomonas in 8.8% and E. coli in 4.4% of the neonates with sepsis [23]. Mondal et al. studied neonatal sepsis among the outborn and inborn babies. He observed that coagulase-negative Staphylococcus, Klebsiella, and Acinetobacter were the commonest causative organisms among inborn babies with sepsis [24]. Kumhar et al. concluded that Klebsiella and Staphylococcus aureus remains the common microorganisms responsible for neonatal sepsis in a tertiary care setting [15]. Swamy et al. conducted a study to correlate sepsis markers and blood culture in neonatal sepsis. They concluded that blood culture was positive in 20.0% of the patients (E. coli being the most commonly isolated organism) [25]. CRP had a high sensitivity of 90.0% and low specificity of 47.0%. Similar results were observed in the present study.

## 5. Strength of the Study

The present study revealed that biochemical tests like CRP, micro-ESR, and gastric aspirate for polymorphs alone or in combination can help in diagnosing early-onset neonatal sepsis.

## 6. Conclusion

Sepsis screening in the neonates is required for detection of infection as blood culture results may be negative, and even positive results may take a few hours. In the present study, the parameters of the sepsis screen were compared with blood culture. C-reactive protein was the most sensitive parameter with a sensitivity of 87.5%. The gastric aspirate for polymorphs and micro-ESR showed high specificity when used alone and in combination.

## Figures and Tables

**Table 1:** Distribution of neonates by sepsis screening parameters.

Parameters	Positive	Negative	Total
TLC	16 (27.0%)	44 (73.0%)	60
CRP	39 (65.0%)	21 (35.0%)	60
Micro-ESR	5 (8.0%)	55 (92.0%)	60
Gastric aspirate	21 (35.0%)	39 (65.0%)	60

**Table 2:** Comparing sepsis screening with blood culture positive neonates.

No. of positive parameters	Blood C/S		Total	Sensitivity	Specificity	PPV	NPV	Accuracy	*p* value
	Positive	Negative							
CRP positive	14	25	39	87.5%	43.1%	35.8%	90.4%	55.0%	0.034
CRP negative	2	19	21						
GA positive	8	13	21	50.0%	70.4%	38.1%	79.4%	65.0%	0.220
GA negative	8	31	39						
Micro-ESR positive	3	2	5	18.7%	95.4%	60.0%	76.3%	75.0%	0.112
Micro-ESR negative	13	42	55						

**Table 3:** Combination of two parameters.

Combination of parameters	Sensitivity	Specificity	PPV	NPV	Accuracy	*p* value
Micro-ESR + GA	12.5%	100%	100%	75.8%	76.6%	0.068
CRP+micro-ESR	18.7%	95.4%	60.0%	76.3%	75.0%	0.112

## Data Availability

No data were used to support this study.
